# Enzymatic Valorization of Whole Blue Crab (*Callinectes sapidus*) Biomass: Optimization of Proteolysis and Characterization of Protein Hydrolysates

**DOI:** 10.3390/foods15111947

**Published:** 2026-06-01

**Authors:** Aniello Falciano, Mariacristina D’Ascoli, Angela Sorrentino, Prospero Di Pierro

**Affiliations:** 1 Department of Agricultural Sciences, University of Naples Federico II, Piazza di Borbone 1, 80055 Portici, Italy; mariacristina.dascoli@unina.it (M.D.); prospero.dipierro@unina.it (P.D.P.); 2 Centre for Food Innovation and Development in the Food Industry, University of Naples Federico II, 80055 Portici, Italy; angela.sorrentino@unina.it

**Keywords:** circular bioeconomy, seafood waste valorization, by-product recovery, degree of hydrolysis, functional food

## Abstract

The Atlantic blue crab (*Callinectes sapidus*) is an invasive species widely distributed in the Mediterranean Sea, causing significant ecological and economic impacts. Despite its low commercial value and the limited utilization of undersized and non-marketable specimens, whole blue crab biomass represents a promising resource for the production of value-added compounds within a circular bioeconomy framework. In this study, whole blue crab biomass, including undersized individuals and non-marketable fractions, was directly valorized through enzymatic hydrolysis for the production of protein hydrolysates. Three commercial proteases (Alcalase, Neutrase, and Papain) were comparatively evaluated for protein hydrolysate production, and the hydrolysis conditions were assessed based on soluble matter yield. The evaluation of hydrolysis conditions identified pH 8, 50 °C, enzyme-to-substrate ratio of 2500 U g^−1^, a solid-to-liquid ratio of 1:4, and a reaction time of 8 h as the most effective conditions for protein solubilization. Under these conditions, maximum soluble matter yields of 57.69% for Alcalase, 51.64% for Neutrase, and 48.44% for Papain were obtained. The obtained hydrolysates were subsequently characterized in terms of protein content and degree of hydrolysis (DH), both of which were significantly affected by enzyme type, following the order Alcalase (64.59 ± 0.75%) > Neutrase (62.29 ± 0.82%) > Papain (58.88 ± 0.65%). A similar trend was observed for degrees of hydrolysis (DH) of the products (43.20 ± 1.24%, 40.29 ± 1.05%, 37.26 ± 1.13%) respectively. Techno-functional properties of the hydrolysates were also enzyme-dependent and closely related to the extent of hydrolysis. Alcalase produced hydrolysates with higher DH, favoring the formation of smaller and more hydrophilic peptides, which enhanced water solubility (98.18 ± 0.51%) and antioxidant activity (77.08 ± 1.06%). In contrast, Papain-derived hydrolysates showed lower hydrolysis extent, likely preserving larger peptide structures and hydrophobic domains associated with higher emulsifying activity (16.10 ± 0.46 m^2^ g^−1^) and foaming capacity (30.47 ± 1.40%). Neutrase displayed intermediate behavior across most parameters. Overall, the results demonstrate that enzymatic hydrolysis of whole blue crab biomass is an effective valorization strategy, and that enzyme selection plays a key role in modulating hydrolysis efficiency and techno-functional properties. This approach provides a sustainable pathway for the management of invasive species while generating functional ingredients for food and nutraceutical applications.

## 1. Introduction

In recent decades, the spread of alien species in European coastal ecosystems has increased markedly due to maritime transport, expanding trade, and climate change [1,2]. The Mediterranean Sea is among the most vulnerable regions to biological invasions, with significant impacts on biodiversity and fisheries-based economies [3,4]. In this context, the Atlantic blue crab *Callinectes sapidus* (Rathbun, 1896), native to the western Atlantic coast, has established stable populations throughout the Mediterranean, where it exhibits invasive behavior [3]. Its ecological plasticity, broad salinity and temperature tolerance, and high reproductive capacity have facilitated the rapid colonization of lagoons, estuaries, and coastal habitats [4]. Recent marine heatwaves have further enhanced habitat suitability, contributing to population expansion [5]. The ecological and economic consequences of this invasion are well-documented. In Mediterranean coastal lagoons, particularly in the northern Adriatic Sea, the rapid proliferation of *Callinectes sapidus* has caused severe impacts on shellfish farming and small-scale fisheries. Recent studies conducted in the Po Delta lagoon reported Manila clam losses reaching up to 100%, with clear evidence of intense predation pressure by blue crab populations on both juvenile and commercial-sized clams [6]. In addition, clam production in some areas declined to near-zero levels during 2024, causing major socioeconomic consequences for local fisheries and aquaculture activities. Recent estimates also reported economic damages exceeding EUR 100 million for the Italian fishery and shellfish sectors associated with blue crab proliferation [7]. Fishery landing analyses and local ecological knowledge have also highlighted increasing disruptions in traditional fishing activities and damage to fishing gear associated with the spread of blue crab populations in Mediterranean coastal areas [4,8]. Targeted fishing and active biomass removal through selective harvesting have therefore been proposed as feasible containment strategies for blue crab populations [3]. However, the long-term sustainability of these management approaches depends on the development of effective biomass valorization pathways capable of converting harvested biomass into value-added products [9]. Although blue crab meat is appreciated in its native range for its sensory and nutritional qualities [10], commercial valorization in the Mediterranean remains limited. Traditional processing yields only 10–15% edible meat [11], generating substantial residual biomass. Moreover, a significant portion of catches consists of undersized individuals that do not meet market standards and are often discarded or diverted to low value uses. From a compositional standpoint, small-sized individuals display a nutritional profile comparable to that of adults, particularly in terms of protein content and amino acid composition. Reported protein levels in crab meat range between 17 and 20% (fresh weight), with a balanced essential amino acid profile [12,13]. Recent analyses of Adriatic populations confirmed consistently high protein levels in muscle tissue regardless of size, along with relevant lipid and mineral fractions [14]. Beyond the edible muscle, whole blue crab biomass including exoskeleton, hepatopancreas, and residual tissues contain substantial structural proteins and bioactive compounds. Arena et al. [11] demonstrated that whole-organism valorization increases overall protein recovery compared to edible fractions alone, highlighting the presence of bioactive peptides and antioxidant compounds in processing residues. The exoskeleton, traditionally regarded as waste, has recently been identified as a source of molecules with reducing and radical-scavenging activity [15]. Similarly, Brai et al. [16] characterized nutraceutical components in Mediterranean blue crab, supporting the concept that entire biomass including non-marketable small individuals represents a valuable biological resource. The recovery and conversion of these protein-rich fractions into functional ingredients therefore requires suitable processing strategies capable of improving biomass utilization and enhancing the accessibility of bioactive compounds. Enzymatic hydrolysis is a sustainable and versatile approach for marine biomass valorization [17]. The degree of hydrolysis (DH) and functional properties of the resulting hydrolysates depend strongly on enzyme type and operating conditions [18,19]. In crustaceans, several proteases have been evaluated. Dey and Dora [20] applied Alcalase to *Penaeus monodon* waste, obtaining hydrolysates with high antioxidant activity. Tkaczewska et al. [21] used Flavourzyme and Protamex on shrimp shells, showing that enzyme selection significantly affects solubility and functional properties. Leiva-Portilla et al. [22] compared Alcalase and Flavourzyme on *Heterocarpus reedi* waste, reporting differences in DH and radical-scavenging capacity. Most of these studies focused on shells, processing residues, and industrial by-products rather than whole biomass. Liu et al. [23] optimized the hydrolysis of marine crab residues using combined commercial proteases, including Alcalase and Flavourzyme, achieving higher DH and improved protein solubility through synergistic endo and exopeptidase activity. Jiang et al. [24] treated shells of *Portunus trituberculatus* with alkaline proteases, generating low-molecular-weight peptides (<3 kDa) with marked antioxidant activity. For *Callinectes sapidus*, Antunes-Valcareggi et al. [25] employed Alcalase to deproteinize processing residues, facilitating chitin and astaxanthin extraction. More recently, Arena et al. [26] compared Alcalase and Protamex on Mediterranean blue crab by-products, demonstrating that enzyme specificity significantly influences DH, peptide molecular distribution, and antioxidant activity. Alcalase produced higher DH values and a greater proportion of low-molecular-weight peptides, whereas Protamex generated distinct peptide profiles with different functional characteristics. Despite these advances, most studies have focused on specific fractions such as shells or processing by-products. The systematic application of enzymatic hydrolysis to whole *C. sapidus* biomass, including both market-sized and undersized individuals, remains largely unexplored, particularly in the context of integrated invasion management and resource valorization. Given that targeted fishing is central to invasion management, developing technologies capable of valorizing the entire harvested biomass, regardless of size, is strategically relevant within a circular bioeconomy framework [27,28]. Accordingly, the present study aimed to evaluate the enzymatic hydrolysis of whole *Callinectes sapidus* biomass using three commercial proteases. The most effective operating conditions were determined based on soluble matter yield, and protein recovery, degree of hydrolysis, and techno-functional properties of the resulting hydrolysates were assessed. This approach supports an integrated strategy in which population control through targeted fishing is coupled with technological valorization, transforming an ecological threat into a high-value resource for food and nutraceutical applications. This may contribute to improving the economic sustainability of biomass removal activities associated with invasive-species control programs.

## 2. Materials and Methods

### 2.1. Materials

Alcalase, Neutrase and Papain enzymes were purchased from Sunson industry group Co., Ltd. (Guangzhou, China).

Hydrochloric acid, sodium hydroxide, boric acid, sulfuric acid (98%), formaldehyde solution (37%), methanol, n-hexane, and other chemicals were purchased from Carlo Erba (Milano, Italy).

### 2.2. Raw Materials and Sample Preparation

Approximately 50 kg of fresh Atlantic blue crab (*Callinectes sapidus*) specimens were obtained in November 2024 from a local fishmonger (Da Leopoldo, Portici, Italy). The sampled population showed size heterogeneity, with a mean body weight of 240 g and individual weights ranging from 50 g to 320 g. Whole specimens were washed thoroughly with tap water to remove external impurities. Aliquots (3 kg) of the cleaned crabs were then homogenized using a buffer solution adjusted to the target pH values employed in the subsequent enzymatic hydrolysis experiments, at a 1:1 (*w*/*w*) ratio. Homogenization was performed using a RoboQbo QB8-4 food processing system (RoboQbo S.r.l., Bologna, Italy) at 100 °C, with a blade speed of 3000 rpm for 10 min under closed-system conditions to minimize moisture loss during heating and to obtain a uniform slurry. The thermal treatment was applied to inactivate endogenous enzymes and standardize the substrate prior to enzymatic hydrolysis. The use of buffered solutions during homogenization ensured immediate pH control and facilitated protein solubilization prior to enzymatic treatment. The resulting homogenate was divided into aliquots and stored at −22 °C until further use.

### 2.3. Proximate Composition of Blue Crabs

The proximate composition of homogenized blue crab samples was determined according to standard AOAC methods [29]. All analyses were performed in triplicate. Moisture content was determined gravimetrically by drying homogenate samples at 105 °C to constant weight. Ash content was measured by incineration in a muffle furnace at 550 °C for 6 h. Total nitrogen was determined using the Kjeldahl method, and crude protein content was calculated using a nitrogen to protein conversion factor of 6.25 [30]. The lipid content was determined by Soxhlet extraction using n-hexane as the solvent. Carbohydrate contents were estimated by difference.

### 2.4. Evaluation Conditions of Enzymatic Assays

The hydrolysis conditions for blue crab (*Callinectes sapidus*) biomass were evaluated by investigating the effects of pH, temperature, reaction time, solid-to-liquid ratio (S/L), and enzyme-to-substrate ratio (E/S). A single-factor experimental approach was adopted to evaluate the individual influence of the main hydrolysis parameters under controlled conditions. Each parameter was investigated at different levels while holding the other variables constant, as shown in Table 1. The study was carried out using three commercial proteases: Alcalase, Neutrase, and Papain. For each hydrolysis assay, approximately 100 g of homogenized sample was mixed with a buffer solution adjusted to the desired pH and brought to the selected solid-to-liquid ratio.

Hydrolysis was performed under constant stirring (170 rpm) at the selected temperature for the specified time. The reaction was initiated by adding the appropriate concentration of enzyme according to the experimental design. Reactions were stopped by heating the samples at 100 °C for 10 min for enzymatic deactivation. The mixtures were then cooled and centrifuged at 15,000 rpm for 10 min at 4 °C (Z 326 K centrifuge, Hermle Labortechnik, Wehingen, Germany) to separate the soluble and insoluble fractions. The supernatant was carefully collected and used to determine the soluble fraction, which was considered the main response variable for optimizing the hydrolysis conditions. Aliquots of the supernatant were dried to constant weight to quantify the soluble fraction. Soluble yield was expressed as the percentage of dry solids recovered in the supernatant relative to the initial sample weight.

### 2.5. Spray Drying of Protein Hydrolysates

Based on the selected hydrolysis conditions, the supernatants obtained from enzymatic hydrolysis were subjected to spray drying to produce protein hydrolysates in powder form (PPH). After centrifugation, the liquid fraction was directly processed in a laboratory-scale spray dryer (BÜCHI Mini Spray Dryer B-191, Büchi Labortechnik AG, Flawil, Switzerland). No carried materials were added during spray drying, and the hydrolysates were dried directly after centrifugation Drying conditions were set according to Abdul-Hamid et al. [31] with slight modifications: feed flow rate of 10 mL min^−1^, aspiration rate of 100%, air flow rate of 700 L h^−1^, inlet temperature of 150 °C and outlet temperature of 80 °C. The resulting powders were collected and stored in airtight containers under cool and dry conditions until further use.

### 2.6. Protein Content (PC) and Degree of Hydrolysis (DH) of PPH

The degree of hydrolysis (DH) was determined according to the method described by Noman et al. [32]: 1 g of PPH was dissolved in 50 mL of distilled water, and the pH was adjusted to 7 with a few drops of NaOH. Then, 10 mL of 37% formaldehyde was added and allowed to react for 5 min at room temperature. The solution was then titrated with 0.1 M NaOH to pH 8.5. Total nitrogen was determined by the Kjeldahl method, while the contents of free amino nitrogen and DH were calculated using Equations (1) and (2):

(1) $$\%free\;amino\;nitrogen=\left[\frac{A\times B\times 14.007}{C\times 1000}\right]\times 100$$

(2) $$DH=[\frac{\%Free\;amino\;nitrogen}{\%total\;nitrogen}]\times 100$$

where A is the volume of NaOH used for titration (mL), B is the molarity of the NaOH solution (0.1 mol L^−1^), 14.007 = atomic weight of nitrogen, and C is the sample weight (g).

Protein content was calculated separately from total nitrogen using a conversion factor of 6.25.

### 2.7. Technological and Functional Proprieties of PPH

#### 2.7.1. Oil Holding Capacity

The oil-holding capacity (OHC) was determined according to the method reported by Rodrìguez-Ambriz et al. [33] with slight modifications. Approximately 1 g of PPH was weighed into a 50 mL Falcon tube and then 10 g of sunflower seed oil was added. The sample was vortexed (uniTEXTER multi, LLG labware, Wilmington, DE, USA) 6 times for 10 s every 5 min. The oil dispersion was then centrifuged at 1000× *g* for 15 min. The amount of oil separated from the hydrolysate was measured, and OHC was calculated as the grams of oil absorbed per gram of PPH protein sample according to Equation (3):

(3) $$OHC=\frac{10\;g\;sunflower\;oil-g\;of\;sunflower\;oil\;separated}{weight\;of\;PPH(g)}$$

#### 2.7.2. Water Solubility Index

The water solubility index (WSI) of PPH was determined by dissolving approximately 1 g of sample in 10 mL of distilled water in a 15 mL Falcon tube. The sample was then incubated at 30 °C for 1 h with constant stirring at 180 rpm, and the dispersion was centrifuged at 15,000× *g* for 10 min. The pellet obtained was dried, and the WSI was determined using Equation (4):

(4) $$WSI(\%)=\frac{weight\;of\;PPH-weight\;of\;pellet(g)}{weight\;of\;PPH(g)}$$

#### 2.7.3. Emulsifying Properties

The emulsification activity index (EAI) and emulsion stability index (ESI) were determined using the methods described by Noman et al. [32]. Specifically, 0.04 g of PPH was dissolved in 40 mL of distilled water in a 100 mL volumetric cylinder. The samples were then mixed with 10 mL of sunflower oil and homogenized (OV5 homogenizer Velp scientifica Srl, Usmate, Italy) for 1 min at 20,000 rpm. After the formation of the emulsion, 50 μL was taken from the bottom and diluted with 5 mL of 0.1% sodium dodecyl sulfate solution. The absorbance of the solutions was measured at 500 nm after 0 and 10 min using UV–Vis (V-730, Jasco, Easton, PA, USA). The EAI and ESI were calculated using Equations (5) and (6):

(5) $$EAI(\frac{m^{2}}{g})=\frac{2\times 2.303\times A\times DF}{l\times C\times ð}$$

(6) $$ESI\left(\%\right)=100-\frac{{EAI}_{0}-{EAI}_{10}}{{EAI}_{10}}\times 100$$

where A = absorption at 500 nm; DF = dilution factor (100); l = path length of cuvette (m); C = protein concentration in aqueous phase (g/m^3^); ð = oil volume fraction (0.25).

#### 2.7.4. Foaming Properties

Foaming capacity (FC) and foam stability (FS) were measured according to the methods of Chel-Guerrero et al. [34] with some modifications. In a 250 mL volumetric cylinder, 2 g of PPH was dissolved in 100 mL of distilled water at room temperature. The sample was homogenized for 2 min at 20,000 rpm. FC was determined by evaluating the volume of foam formed after 2 min of homogenization. FS was determined by evaluating the decay of foam volume over time from 1 to 4 min. FC and FS were calculated using Equations (7) and (8):

(7) $$FC(\%)=\frac{V_{2}-V_{1}}{V_{1}}\times 100$$

(8) $$FS(\%)=\frac{Foam\;volume\;after\;time}{Initial\;foam\;volume}\times 100$$

where V_1_ = volume before whipping; V_2_ = volume after whipping.

All functional properties were evaluated using the same protein concentration in order to ensure direct comparison among hydrolysates.

#### 2.7.5. Antioxidant Activity

Antioxidant activity was evaluated using the DPPH radical scavenging assay according to Bautista-Espinoza et al. [35] with slight modifications. A methanolic solution of DPPH (0.1 mM) was freshly prepared. PPH was dissolved in distilled water to a final concentration of 0.1 g mL^−1^. An aliquot of 50 μL of the sample solution was added to 950 μL of DPPH solution and vortexed for 30 s. The mixtures were then incubated in the dark at room temperature for 1 h. Absorbance was measured at 517 nm using a UV–Vis spectrophotometer (V-730, Jasco, Easton, PA, USA). The radical scavenging activity was expressed as the percentage inhibition of DPPH radical and was calculated using Equation (9):

(9) $$\mathrm{DPPH}\cdot \mathrm{scavenging}\;\mathrm{activity}\;(\%)\;=\frac{\left(Ab-As\right)}{Ab}\times 100$$

where Ab = absorbance of the blank sample; As = absorbance of the extract.

### 2.8. Statistical Analysis

All analyses were performed in triplicate, and results are expressed as mean ± standard deviation. Differences among treatments were evaluated by one-way analysis of variance (ANOVA), considering enzyme type (Alcalase, Neutrase, and Papain) as the main factor. When significant differences were detected (*p* < 0.05), Tukey’s HSD post hoc test was applied for multiple comparisons of means. Statistically significant differences are indicated by different letters within the same row. Statistical analyses were performed using Python (version 3.14).

## 3. Results and Discussion

### 3.1. Proximate Composition

The proximate composition of blue crab (*Callinectes sapidus*) biomass, expressed on a dry weight basis, is presented in Table 2. The results showed that the samples were mainly composed of mineral (35.00 ± 2.99%), carbohydrates (34.14 ± 0.20%), and proteins (30.42 ± 0.12%), while the lipid content was negligible (0.44 ± 0.11%). This distribution reflects the composite nature of the biomass, in which both organic and structural components contribute significantly to the overall composition. In this study, the entire organism, including edible tissues and the exoskeleton, was analyzed using a mixed batch of specimens of different sizes, including undersized individuals not intended for commercial sale. This approach provides a realistic representation of the available biomass and allows for an assessment of its overall suitability for protein recovery processes. Despite the high proportion of mineral and structural components, the protein content (30.42% on a dry weight basis) remains substantial, confirming the potential of the whole blue crab biomass as a source of recoverable proteins. The elevated ash and carbohydrate fractions are primarily associated with the exoskeleton, which is rich in calcium carbonate and structural polysaccharides such as chitin. The values obtained are not directly comparable with most of the literature data, which typically refer to specific anatomical fractions rather than the whole organism. Previous studies have mainly focused on edible tissues, reporting protein contents on a fresh weight basis of 21.4–22.45% for crab meat [36] and 18.79–19.11% for different anatomical parts [37]. Similarly, Farragut [38] reported protein values ranging from 10% to 19% depending on the fraction analyzed. In addition, Fuso et al. [14] highlighted a marked compositional heterogeneity between tissues and carapace, with mineral content reaching up to 65% (dry weight) in the latter. Overall, the compositional profile observed in this study reflects the integration of all anatomical components and provides a comprehensive characterization of the biomass. These results support the use of whole *C. sapidus*, including non-marketable individuals, as a suitable substrate for enzymatic hydrolysis and protein recovery applications.

### 3.2. Optimization of Enzymatic Hydrolysis Conditions

**Figure 1:**
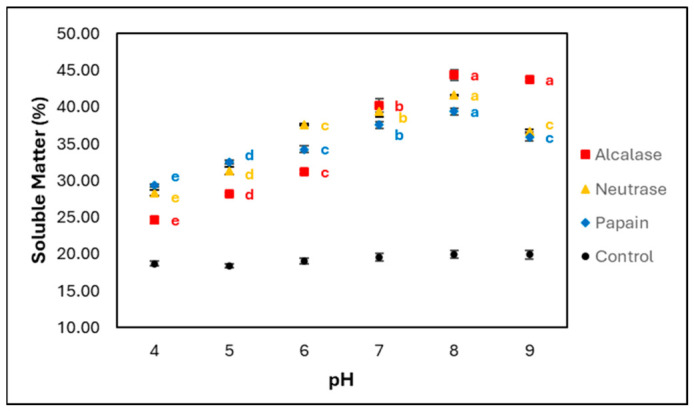
Effect of pH on soluble matter yield (%) during enzymatic hydrolysis of blue crab homogenate using three different enzymes: Alcalase, Neutrase, and Papain. Data are expressed as mean ± standard deviation (n = 3). For each enzyme, different letters indicate statistically significant differences among the tested conditions according to Tukey’s HSD test (*p* < 0.05).

**Figure 2:**
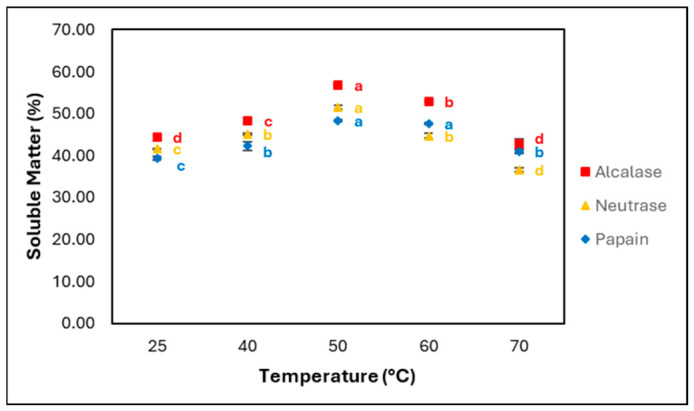
Effect of temperature (°C) on soluble matter yield (%) during enzymatic hydrolysis of blue crab homogenate using three different enzymes: Alcalase, Neutrase, and Papain. Data are expressed as mean ± standard deviation (n = 3). For each enzyme, different letters indicate statistically significant differences among the tested conditions according to Tukey’s HSD test (*p* < 0.05).

**Figure 3:**
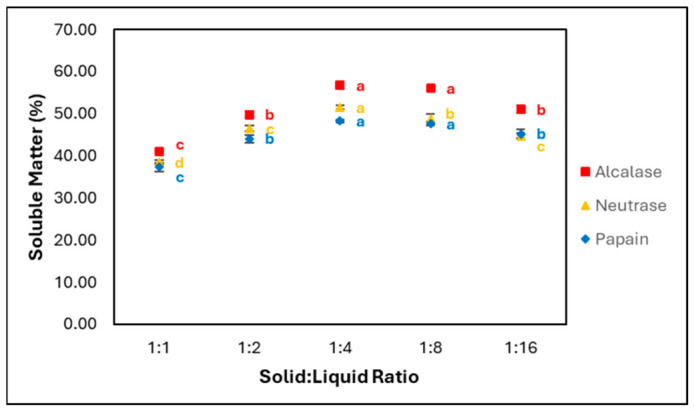
Effect of solid:liquid ratio on soluble matter yield (%) during enzymatic hydrolysis of blue crab homogenate using three different enzymes: Alcalase, Neutrase, and Papain. Data are expressed as mean ± standard deviation (n = 3). For each enzyme, different letters indicate statistically significant differences among the tested conditions according to Tukey’s HSD test (*p* < 0.05).

**Figure 4:**
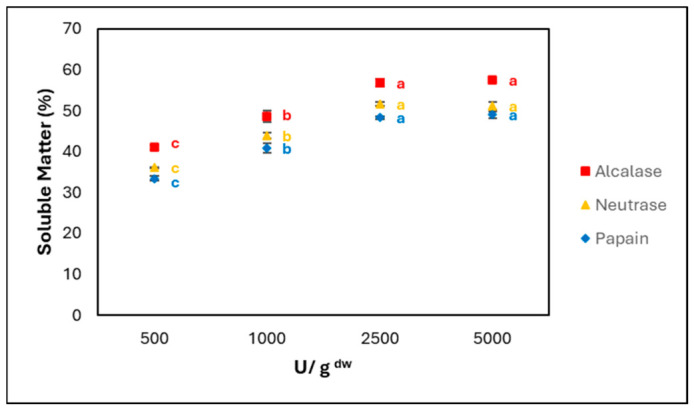
Effect of enzyme concentration (U/g) on soluble matter yield (%) during enzymatic hydrolysis of blue crab homogenate using three different enzymes: Alcalase, Neutrase, and Papain. Data are expressed as mean ± standard deviation (n = 3). For each enzyme, different letters indicate statistically significant differences among the tested conditions according to Tukey’s HSD test (*p* < 0.05).

**Figure 5:**
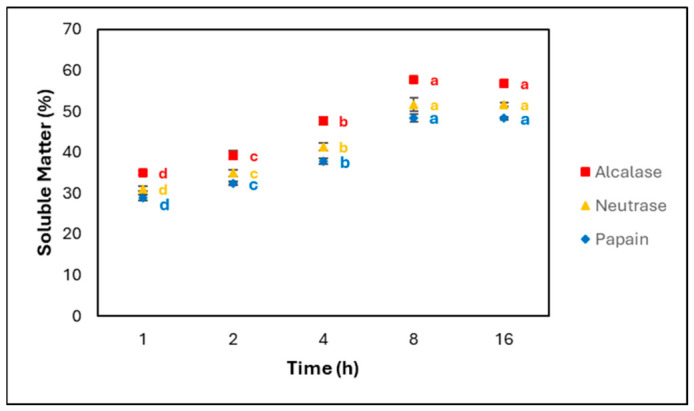
Effect of time (h) on soluble matter yield (%) during enzymatic hydrolysis of blue crab homogenate using three different enzymes: Alcalase, Neutrase, and Papain. Data are expressed as mean ± standard deviation (n = 3). For each enzyme, different letters indicate statistically significant differences among the tested conditions according to Tukey’s HSD test (*p* < 0.05).

The efficiency of enzymatic hydrolysis is strongly influenced by the operating conditions of the reaction system. Parameters such as pH, temperature, solid-to-liquid ratio, enzyme concentration, and reaction time affect both protease activity and substrate accessibility, thereby determining the extent of protein solubilization [17]. In addition, enzyme specificity plays an important role in determining the extent of protein cleavage, peptide size distribution, and the balance between hydrophilic and hydrophobic residues exposed during hydrolysis, ultimately affecting the functional characteristics of the resulting hydrolysates. The effect of these variables on the hydrolysis of blue crab biomass is shown in Figure 1, Figure 2, Figure 3, Figure 4 and Figure 5.

Under the test conditions (time: 16 h; Temperature: 25 °C; S/L ratio: 1:4; E/S: 2500 U/g^dw^), the pH of the reaction medium had a significant effect on protein solubilization (Figure 1), with an increase in soluble matter observed as conditions shifted from acidic to neutral and slightly alkaline. The most pronounced increase occurred between pH 6 and pH 8, where the highest values were recorded for all enzymes. Among the proteases tested, Alcalase showed the highest solubilization capacity over the entire pH range, reaching 44.35% at pH 8, while Neutrase and Papain showed lower yields but followed a similar trend. A slight stabilization or decrease in yield was observed at pH 9. Although the differences between pH 8 and pH 9 were relatively limited, pH 8 was selected as the most suitable condition since it consistently provided the highest soluble matter values for all enzymes without further improvements under more alkaline conditions. In addition, the control samples showed soluble matter values of approximately 18–20%, indicating that enzymatic hydrolysis substantially enhanced biomass solubilization compared to non-enzymatic conditions. This behavior reflects the combined effect of pH on protein structure and enzyme activity. Alkaline proteases such as Alcalase typically exhibit optimal activity under mildly alkaline conditions, where the ionization state of catalytic residues enhances peptide bond cleavage [17]. At alkaline pH, deprotonation of amino acid side chains may also contribute to partial unfolding of protein structures, increasing the exposure of buried peptide bonds to enzymatic attack. Under acidic conditions, reduced ionization of catalytic residues and lower substrate accessibility may limit proteolytic efficiency. In addition, alkaline conditions promote the dissociation of protein–chitin complexes in crustacean matrices, increasing the accessibility of proteolytic cleavage sites [39]. This effect is particularly relevant in whole blue crab biomass, where proteins are embedded within a complex matrix containing minerals, chitin, connective tissues, and membrane components that may sterically hinder enzyme accessibility. Similar optimal pH ranges (7–9) have been reported for the hydrolysis of fish and crustacean by-products [40,41]. Overall, the results indicate that pH 8 is the most favorable condition for enzymatic hydrolysis of blue crab biomass under the conditions tested.

Figure 2 shows the effect of temperature on the hydrolysis yield at the following assay conditions: time 16 h, S/L ratio 1:4, E/S 2500 U/g^dw^, and pH 8. Increasing the temperature from 25 °C to 50 °C resulted in a progressive increase in soluble matter for all enzymes. Maximum solubilization was observed at 50 °C, with values of 56.75% for Alcalase, 51.61% for Neutrase, and 48.30% for Papain. At higher temperatures, a decrease in yield was observed, particularly at 70 °C. The increase up to 50 °C can be attributed to enhanced molecular mobility and a higher frequency of enzyme–substrate interactions, which promote hydrolysis [42]. Temperature increase may also favor conformational relaxation of protein aggregates, facilitating enzyme penetration into the substrate matrix and improving accessibility of cleavage sites. Increasing temperature may also reduce the viscosity of the homogenized biomass, improving mass transfer and substrate dispersion within the reaction system. In contrast, further temperature increases likely caused partial thermal denaturation of the enzymes, leading to reduced catalytic activity [17]. This behavior is consistent with the known thermal stability of commercial proteases used for marine protein hydrolysis, which generally maintain high catalytic activity at moderate temperatures but may progressively lose stability and catalytic efficiency after prolonged exposure to higher temperatures, in agreement with the trends observed in the present study. Excessive temperatures may additionally induce protein aggregation phenomena through hydrophobic interactions, reducing the availability of soluble substrates for enzymatic attack. Although commercial proteases such as Alcalase and Papain are relatively thermostable, prolonged exposure to elevated temperatures may also promote irreversible conformational modifications affecting enzyme functionality. These results are consistent with previously reported optimal temperature ranges (45–55 °C) for enzymatic hydrolysis of marine biomass, including fish and crustacean by-products treated with Alcalase and other commercial proteases [22,41,42].

The solid-to-liquid ratio had a significant effect on the hydrolysis yield (Figure 3). The conditions set for the assay were: time: 16 h; E/S: 2500 U/g^dw^, pH 8, and temperature 50 °C. As the ratio was increased from 1:1 to 1:4, an increase in soluble matter was observed for all enzymes. Maximum solubilization was observed at a ratio of 1:4. Further increases in solvent volume did not improve the yield, and a decrease was observed at higher ratios, such as 1:16. This behavior reflects the balance between improved mass transfer at moderate dilution and reduced enzyme–substrate interactions at excessive dilution. While increasing the amount of solvent enhances substrate dispersion, excessive dilution lowers the effective concentrations of both enzyme and substrate, limiting hydrolysis efficiency [22]. At low solvent volumes, the high viscosity of the homogenized crab biomass may also reduce enzyme diffusion and limit contact between proteases and substrate proteins. Moreover, insufficient hydration may reduce protein swelling and limit solvent penetration into the substrate matrix, decreasing the accessibility of peptide bonds to proteolytic attack. Similar trends have been reported for enzymatic hydrolysis of marine biomass, where intermediate solid-to-liquid ratios (1:3–1:5) are generally considered optimal for maximizing protein solubilization [10,41]. From an application perspective, moderate S/L ratios are also advantageous because they reduce water consumption and downstream concentration costs while maintaining high hydrolysis efficiency, which is relevant for the economic sustainability of potential industrial-scale applications.

Figure 4 shows the effect of enzyme concentration when the assay was set to the following conditions: time: 16 h; pH 8; temperature 50 °C; and S/L ratio: 1:4. By increasing the concentration from 500 to 2500 U g^−1^, a significant increase in soluble matter was observed for all enzymes, reaching values of 56.75% for Alcalase, 51.60% for Neutrase, and 48.30% for Papain. No further significant improvement was observed when the concentration was increased to 5000 U g^−1^. This behavior may be attributed to substrate saturation. At high enzyme concentrations, the availability of substrate becomes the limiting factor, and additional enzyme does not lead to increased hydrolysis rates [43]. The plateau observed at higher enzyme dosages suggests that most accessible peptide bonds had already been hydrolyzed under the tested conditions. At this stage, residual proteins may become progressively less accessible due to steric hindrance, aggregation phenomena, or association with insoluble chitin-rich fractions. Similar trends have been reported in the enzymatic hydrolysis of fish by-products, where increasing enzyme concentration beyond an optimal level does not result in further improvements in solubilization yield [40,41]. These results indicate that increasing enzyme dosage beyond a certain threshold may not be economically advantageous for industrial applications.

Finally, Figure 5 shows the effect of reaction time under the assay conditions of pH 8, a temperature of 50 °C, an S/L ratio of 1:4, and an E/S ratio of 2500 U/g^dw^. The soluble matter increased significantly over time, reaching maximum values after 8 h: 57.69% for Alcalase, 51.64% for Neutrase, and 48.44% for Papain. These values were not significantly different from those observed at 16 h. Therefore, an 8 h duration was selected in order to reduce process duration and operational costs while maintaining comparable hydrolysis yields. This behavior is indicative of the typical kinetics associated with enzymatic hydrolysis. In the initial phase, the reaction rate is high due to the abundance of available substrate. As hydrolysis proceeds, the concentration of intact proteins decreases, and the system approaches a plateau. Furthermore, the accumulation of peptides has been demonstrated to contribute to product inhibition, thereby further limiting the reaction rate [17,43]. The accumulation of soluble peptides and amino acids may also modify the physicochemical environment of the reaction medium, potentially affecting enzyme–substrate affinity and catalytic efficiency over time. The plateau that was observed after 8 h of incubation indicates that the hydrolysis system has gradually achieved equilibrium between peptide bond cleavage and substrate availability. These findings are in accordance with the findings of previous studies, which reported optimal hydrolysis times ranging from 6 to 10 h for marine protein hydrolysates [22,44].

Based on the optimization results (Figure 1, Figure 2, Figure 3, Figure 4 and Figure 5), enzymatic hydrolysis of blue crab biomass was carried out under the identified optimal conditions: pH 8, 50 °C, solid-to-liquid ratio of 1:4, enzyme concentration of 2500 U/g^dw^ and reaction time of 8 h. Subsequent to hydrolysis and separation of the insoluble fraction, the supernatants were recovered and subjected to spray drying. The resulting powders were subsequently characterized in terms of process yield, protein content, and degree of hydrolysis.

### 3.3. Protein Content and Degree of Hydrolysis of PPH

Protein content (PC) and the degree of hydrolysis (DH) of the obtained protein hydrolysate powder (PPH) are reported in Table 3. Both parameters were significantly influenced by the enzyme type (*p* < 0.05), following the order Alcalase > Neutrase > Papain.

Alcalase yielded the highest PC (64.59 ± 0.75%) and DH (43.20 ± 1.24%), followed by Neutrase (PC: 62.29 ± 0.82%; DH: 40.29 ± 1.05%) and Papain (PC: 58.88 ± 0.65%, DH: 37.26 ± 1.13%). This result confirms the strong relationship between the extent of hydrolysis and protein solubilization, as increased fragmentation enhances the transfer of proteins into the liquid phase [45]. Since DH reflects the extent of peptide bond cleavage, these results indicate that Alcalase promoted a more extensive proteolytic degradation of *Callinectes sapidus* proteins. The higher DH observed for Alcalase may be attributed to its broad substrate specificity and strong endoprotease activity [45], which likely promoted a more extensive cleavage of peptide bonds and the formation of more soluble peptide fractions. This extensive fragmentation may also increase the exposure of polar and ionizable amino acid residues, favoring protein solubilization and transfer into the aqueous phase. In contrast, the lower DH values observed for Papain suggest a less extensive hydrolysis process, potentially resulting in the preservation of larger peptide structures and partially hydrolyzed protein fragments. Such structures may retain greater conformational flexibility and amphiphilic balance, properties that are often associated with improved interfacial functionality. Similar trends have been widely reported in the literature. Chalamaiah et al. [46] observed a DH of 62% for Alcalase-treated fish egg proteins compared to 17% for Papain, while Noman et al. [32] reported DH values of 25% for Papain in sturgeon muscle. In crustacean matrices, Antunes-Valcareggi et al. [25] showed that Alcalase hydrolyzed approximately 30% of total proteins in blue crab waste within 120 min. More generally, Ovissipour et al. [43] reported DH values ranging from 13% to 46% for marine protein hydrolysis, highlighting the strong influence of process conditions. The intermediate behavior observed for Neutrase suggests a moderate hydrolytic action, producing peptide fractions with intermediate functional and structural characteristics between those generated by Alcalase and Papain. Overall, the results demonstrate that enzyme selection plays a key role in determining both hydrolysis efficiency and protein recovery. Considering the complex composition of whole blue crab biomass, including shell-derived fractions and insoluble structural components, the obtained DH and protein recovery values indicate an effective enzymatic solubilization of the available protein fraction. Under the investigated conditions, Alcalase proved to be the most effective enzyme for blue crab protein hydrolysis, achieving DH and protein content values within the upper range reported for marine protein hydrolysates.

### 3.4. Technological and Functional Properties

The technological and functional properties of the protein hydrolysate powders (PPH) obtained with Alcalase, Neutrase, and Papain are presented in Table 4. The enzyme type significantly affected all of the analyzed parameters (*p* < 0.05), resulting in different functional characteristics.

The water solubility index (WSI) was very high in all samples, with values of 98.18 ± 0.51% for Alcalase, 95.40 ± 0.72% for Neutrase, and 91.88 ± 1.08% for Papain. Overall, the PPH exhibited excellent water solubility (>91%) regardless of the enzyme used. Alcalase showed slightly higher values, which may be associated with its higher DH and the formation of smaller and more hydrophilic peptides, enhancing protein–water interactions. The extensive hydrolysis promoted by Alcalase may have increased the exposure of charged and polar amino acid residues, improving hydration capacity and peptide–water interactions. The very high solubility observed for all hydrolysates suggests that enzymatic treatment effectively disrupted the native protein matrix of whole blue crab biomass, generating peptides with strong affinity for the aqueous phase. Similar solubility values (>90%) have been widely reported for fish protein hydrolysates at high DH [47,48].

Conversely, properties related to interfacial behavior, including oil holding capacity (OHC), emulsifying activity (EAI), and foaming capacity (FC), followed an opposite trend. OHC increased from 1.80 ± 0.07 g g^−1^ (Alcalase) to 2.05 ± 0.10 g g^−1^ (Neutrase) and 2.38 ± 0.09 g g^−1^ (Papain). Similarly, EAI increased from 13.12 ± 0.28 m^2^ g^−1^ (Alcalase) to 14.35 ± 0.33 m^2^ g^−1^ (Neutrase) and 16.10 ± 0.46 m^2^ g^−1^ (Papain), while FC increased from 20.00 ± 0.92% (Alcalase) to 24.47 ± 0.91% (Neutrase) and 30.47 ± 1.40% (Papain). This behavior suggests that lower DH values, associated with less extensive hydrolysis, favor the retention of structural features that enhance interactions with oil phases and adsorption at interfaces. In contrast, the higher DH observed for Alcalase likely leads to the formation of more hydrophilic components, resulting in reduced interfacial activity. Excessive hydrolysis may reduce molecular size and structural flexibility to the point that peptides become less effective in forming cohesive interfacial films around air or oil droplets. The improved interfacial and foaming properties observed for Papain hydrolysates may be associated with the presence of larger and more amphiphilic peptide structures generated under lower hydrolysis intensity. These structures may retain both hydrophobic domains capable of interacting with non-polar phases and hydrophilic regions able to stabilize aqueous interfaces. These findings are consistent with previous studies indicating that moderate hydrolysis and higher hydrophobicity improve interfacial properties [17,41,49], with comparable OHC values (1.5–2.5 g g^−1^) reported for fish protein hydrolysates [50,51].

Despite these differences, the emulsion stability index (ESI) remained consistently high across all samples (96.47–99.80%), indicating that all hydrolysates were able to maintain stable emulsions. The limited differences among treatments suggest that despite variations in EAI, sufficient structural integrity was retained to prevent coalescence, in agreement with previous findings (>90%) for fish protein hydrolysates [51]. Similarly, foam stability (FS) showed relatively small variations, ranging from 89.54 ± 1.37% to 94.88 ± 1.11%, confirming that once the interfacial layer was formed, all samples were able to maintain stable systems. These results suggest that hydrolysis intensity mainly affected interface formation rather than the long-term stability of the systems once formed.

Antioxidant activity, evaluated by DPPH radical scavenging, followed a trend similar to solubility, with values of 77.08 ± 1.06% for Alcalase, 73.60 ± 1.11% for Neutrase, and 69.40 ± 0.92% for Papain. This suggests that higher DH values favor the formation of bioactive peptides with enhanced radical scavenging activity, likely due to the increased exposure of functional amino acid residues. The higher antioxidant activity associated with Alcalase hydrolysates may also reflect the greater release of peptide fractions capable of hydrogen donation, electron transfer, and radical stabilization. In particular, the exposure of amino acid residues such as histidine, tyrosine, methionine, cysteine, and aromatic residues may contribute to electron transfer reactions and free radical neutralization. Similar values (65–80%) have been reported for fish protein hydrolysates [41,52], and the role of low-molecular-weight peptides in antioxidant mechanisms is well-documented [53].

The results obtained demonstrate a discernible correlation between the extent of hydrolysis and the functional performance of the samples. Higher DH values, as observed for Alcalase, promoted solubility and antioxidant activity, whereas lower DH values favored interfacial and foaming properties. These observations highlight the dual effect of hydrolysis intensity on functionality: extensive hydrolysis enhances peptide solubilization and bioactivity, whereas moderate hydrolysis better preserves amphiphilic structures involved in interface stabilization. From a biochemical perspective, controlling hydrolysis intensity appears essential to modulate the balance between peptide fragmentation, surface activity, and biofunctional properties. Overall, the results demonstrate that enzyme selection can be effectively used to tailor the functional characteristics of protein hydrolysates according to the intended application, with higher DH favoring solubility and antioxidant activity and lower DH preserving emulsifying and foaming performance.

## 4. Conclusions

This study demonstrated that whole blue crab (*Callinectes sapidus*) biomass, including non-marketable individuals, represents a suitable substrate for the production of protein hydrolysates through enzymatic processing. The evaluation of hydrolysis conditions allowed for the selection of suitable operating parameters for effective protein solubilization and hydrolysate recovery from whole crab biomass. The results also confirmed that enzyme selection strongly influences both hydrolysis efficiency and the techno-functional characteristics of the obtained hydrolysates. Differences in the degree of hydrolysis among the tested enzymes were associated with distinct functional profiles, particularly in terms of solubility, antioxidant activity, and interfacial properties. These findings demonstrate that enzyme selection plays a key role in determining the functional characteristics of protein hydrolysates and may therefore support their use in different food and nutraceutical applications. Overall, this approach represents a sustainable strategy for the valorization of whole blue crab biomass within a circular bioeconomy framework, contributing to the management of an invasive species while generating value-added functional ingredients.

## Figures and Tables

**Table 1:** Experimental design used for the evaluation of enzymatic hydrolysis conditions of *Callinectes sapidus* biomass. Each parameter was evaluated at different levels while keeping the others constant.

Parameter Studied	Fixed Conditions	Evaluated Conditions
pH	25 °C; 1:4; 2500 U/g^dw^; 16 h	4, 5, 6, 7, 8, 9
Temperature (°C)	1:4; 2500 U/g^dw^; 16 h; *pH*	25, 40, 50, 60, 70
Solid/liquid ratio (S/L)	2500 U/g^dw^; 16 h; *pH*; *T*°	1:1, 1:2, 1:4, 1:8, 1:16
Enzyme/substrate (U/g^dw^)	16 h; *pH*; *T*°; *S/L*	500, 1000, 2500, 5000
Time (h)	*pH*; *T*°; *S/L*; *E/S*	1, 2, 4, 8, 16

Italicized parameters indicate the optimal conditions identified for each enzyme.

**Table 2:** Proximate composition of *Callinectes sapidus* expressed on a dry weight basis. Values are reported as mean ± standard deviation (n = 3).

Components (%)	g/100 g^dw^
Ash	35.00 ± 2.99
Proteins	30.42 ± 0.12
Lipids	0.44 ± 0.11
Carbohydrates	34.14 ± 0.20

**Table 3:** Protein content (PC) and degree of hydrolysis (DH) of protein hydrolysate powder (PPH) obtained using different enzymes. Values are expressed as mean ± standard deviation (n = 3). Different superscript letters within the same row indicate significant differences among hydrolysates, as determined by one-way ANOVA followed by Tukey’s HSD post hoc test (*p* < 0.05).

Parameter	Alcalase	Neutrase	Papain
PC (%)	64.59 ± 0.75 ^a^	62.29 ± 0.82 ^b^	58.88 ± 0.65 ^c^
DH (%)	43.20 ± 1.24 ^a^	40.29 ± 1.05 ^b^	37.26 ± 1.13 ^c^

**Table 4:** Technological and functional properties of PPH obtained with different enzymes. Values are expressed as mean ± standard deviation (n = 3). Different letters within the same row indicate significant differences between hydrolysates as determined by one-way ANOVA followed by Tukey’s HSD post hoc test (*p* < 0.05). WSI: water solubility index; OHC: oil holding capacity; EAI: emulsifying activity index; ESI: emulsion stability index; FC: foaming capacity; FS: foam stability; DPPH: free radical scavenging activity.

Parameters	Alcalase	Neutrase	Papain
WSI (%)	98.18 ± 0.51 ^a^	95.40 ± 0.72 ^b^	91.88 ± 1.08 ^c^
OHC (g/g)	1.80 ± 0.07 ^c^	2.05 ± 0.10 ^b^	2.38 ± 0.09 ^a^
EAI (m^2^/g)	13.12 ± 0.28 ^c^	14.35 ± 0.33 ^b^	16.10 ± 0.46 ^a^
ESI (%)	96.47 ± 1.07 ^b^	98.20 ± 0.72 ^ab^	99.80 ± 0.90 ^a^
FC (%)	20.00 ± 0.92 ^c^	24.47 ± 0.91 ^b^	30.47 ± 1.40 ^a^
FS (%)	89.54 ± 1.37 ^b^	92.50 ± 1.25 ^ab^	94.88 ± 1.11 ^a^
DPPH (%)	77.08 ± 1.06 ^a^	73.60 ± 1.11 ^b^	69.40 ± 0.92 ^c^

## Data Availability

The original contributions presented in this study are included in the article. Further inquiries can be directed to the corresponding author.
